# Understanding the endo-lysosomal network in neurodegeneration

**DOI:** 10.1098/rstb.2022.0372

**Published:** 2024-04-08

**Authors:** Peter J. Cullen, Henne Holstege, Scott A. Small, Peter St George-Hyslop

**Affiliations:** ^1^ University of Bristol School of Biochemistry, Bristol BS8 1QU, UK; ^2^ Genomics of Neurodegenerative Diseases and Aging, Vrije Universiteit Amsterdam, Amsterdam, The Netherlands; ^3^ Department of Neurology, Columbia University, New York, NY 10032, USA; ^4^ Department of Medicine (Division of Neurology), Temerty Faculty of Medicine, University Health Network, University of Toronto, Toronto, Canada, M5T 0S8

**Keywords:** endosomes, lysosomes, neurodegeneration, Retromer, Alzheimer's disease, Parkinson's disease

Neurodegenerative diseases represent one of the world's most pressing socio-economic burdens, the cost of which are predicted to increase within an ageing population. The symptoms of neurodegenerative diseases are variable, ranging from dysfunction in motor control and mood behaviour through to cognitive decline and dementia. Currently, there are no cures and treatment is through care. A major goal of neurodegenerative research is to achieve an understanding of the causes, mechanisms and progression of these diseases [1].

Integral proteins and their associate proteins and lipids (collectively termed ‘cargo’) are actively sorted and trafficked through the multiple compartments of the endosomal pathway—which includes the early, recycling, and late endosomes and the degradative lysosome [2]. Over the last 10 years significant strides have been made in identifying and characterizing the molecular machinery that orchestrate the complex processes of cargo sorting through the endosomal pathway [3,4]. Multi-protein assemblies, such as the Retromer and Retriever complexes, have paved the way for an intensification in the mechanistic analysis and functional dissection of this pathway.

In parallel, genomic analysis of Alzheimer's disease, Parkinson's disease and related neurodegenerative disorders has identified that perturbation in these complexes and the endosomal pathway more generally is a common feature across these diseases [5,6]. A complementary series of studies in model systems have clarified the specific mechanisms of these genomic abnormalities. The brain therefore turns out to be particularly vulnerable to defects in the endosomal pathway.

Collectively, a clear picture is beginning to emerge that the endosomal pathway plays a vital neuroprotective role in neuronal homeostasis [1]. Importantly, these discoveries have raised several important and timely questions: why is the brain particularly vulnerable to endosomal pathway defects? How do endosomal pathway defects lead to disease? How is it that subtly different defects in the endosomal pathway lead to one type of neurodegenerative disease or the other? Can endosomal pathway defects be targeted therapeutically?

This issue encompasses a series of reviews and commentaries from leaders in this emerging field. It stems from a Hooke Discussion meeting held at the Royal Society in November 2022 (COVID postponed from 2020), a full video playlist of which can be found at: https://royalsociety.org/science-events-and-lectures/2022/11/endosomal-network/.

## Data Availability

This article has no additional data.
